# Zhengqing fengtongning sustained-release tablets prevents gout flares in the process of ULT: A randomized, positive control, double-blind, double-simulation, multicenter trial

**DOI:** 10.1097/MD.0000000000029199

**Published:** 2022-05-13

**Authors:** Ertao Jia, Shasha Hu, Hongling Geng, Haiqiong Zhu, Jingjing Xie, Yuya Xiao, Yubao Jiang, Min Xiao, Jianyong Zhang

**Affiliations:** a The Department of Rheumatology, Shenzhen Traditional Chinese Medicine Hospital, Shenzhen, Guangdong, China; b The Department of Rheumatology, the fourth Clinical Medical College of Guangzhou University of Chinese Medicine, Shenzhen, Guangdong, China; c Shenzhen Traditional Chinese Medicine Hospital Affiliated to Nanjing University of Chinese Medicine, Shenzhen, Guangdong, China; d The Department of Gynecology, Guangdong Provincial Hospital of Chinese Medicine, the Second Affiliated Hospital of Guangzhou University of Chinese Medicine, Guangzhou, Guangdong, China.

**Keywords:** acute gout flares, administrative information, sinomenine, uric acid

## Abstract

**Introduction:**

When initiating urate-lowering therapy, using anti-inflammatory prophylaxis therapy for at least 3 to 6 months is strongly recommended. Previous studies have found that zhengqing fengtongning sustained-release tablets (sinomenine) can improve inflammation in the acute phase of gout; however, the efficacy of urate-lowering therapy in reducing frequency of acute flares still needs to be investigated. The aim of the present study is to explore the efficacy and safety of sinomenine for prophylaxis of acute flares when initiating urate-lowering therapy.

**Methods and analysis:**

This randomized, placebo-controlled, double-blinded trial will include a total of 210 gout patients who meet the study criteria. The patients will be randomized (1:1) to the test group and the control group. The intervention is planned to be performed for 12 weeks with a follow-up of 12 weeks. All patients would be administered febuxostat (40 mg/d) and concomitant anti-inflammatory prophylaxis therapy. Sinomenine and colchicine placebo are administered in the sinomenine group, sinomenine placebo and colchicine are administered in the colchicine group. The primary outcome is the rate of acute gout flares in subjects within 12 weeks of the treatment period. The secondary outcomes include the times of acute gout flares and the duration of each acute flares within 12 weeks; the compliance rate in patients whose UA levels ≤6.0 mg/dL (360 μmol/L) at the weekend of 2nd, 4th, 8th, and 12th week in each group; the proportion of patients with ≥1 and ≥2 gout flares within 12 weeks; average visual analogue scale/score pain score during gout flares; and the oral dose of etoricoxib will be used to control the onset of acute flares within 12 weeks.

**Ethics and dissemination:**

The Institutional Medical Ethics Committee have approved the trial protocol. We plan to publish the results of this study in a peer-reviewed journal.

**Trial registration:**

ChiCTR, ChiCTR2100045114, Registered 8 April 2021 http://www.chictr.org.cn/showproj.aspx?proj=124688

## 1 Introduction

### 1.1 Background and rationale

Gout is a painful form of arthritis mainly caused by monosodium urate crystals formed during prolonged hyperuricemia.^[1,2]^ Clinical guidelines for gout treatment suggest the use of long-term urate-lowering therapy (ULT) to reduce serum uric acid (SUA) level <6.0 mg/dL and to prevent gouty flares and complications by dissolving the pathogenic crystals.^[3,4]^ As the first few months of ULT may increase the risk of acute gout flares.^[3,5]^ Prophylaxis against acute flares over the initial 3 to 6 months period of ULT can be further enhanced with concomitant colchicine or nonsteroidal anti-inflammatory drugs (NSAIDs).^[3,6]^

However, the patients with gout often have kidney disease, cardiovascular disease, diabetes, and other diseases,^[7]^ which limits the use of prophylaxis drug. Sinomenine is the main active ingredient extracted from traditional antirheumatic medicine sinomenium actum. Previous studies have found that sinomenine has anti-inflammatory properties.^[8,9]^ Our observational study has found that sinomenine could improve inflammation in the acute phase of gout; however, its efficacy for prophylaxis of acute flares when initiating urate-lowering therapy needs to be further investigated.

### 1.2 Objectives

The aim of the present study is to explore the efficacy and safety of sinomenine for prophylaxis of acute flares when initiating urate-lowering therapy.

### 1.3 Trial design

This randomized, placebo-controlled, double-blinded, double-simulation, multicenter trial will be completed in 7 research centers including Shenzhen Hospital of Traditional Chinese Medicine over 12 weeks. A total of 210 gout patients who meet the study criteria will be randomized (1:1) to the test group and the control group. The intervention will last for 12 weeks with a follow-up of 12 weeks. Informed written consent was obtained prior to the commencement of this study. All enrolled subjects will be fully informed about the purpose, process, and possible risks of the study.

## 2 Methods: participants, interventions, and outcomes

### 2.1 Study setting

The study will be completed in 7 research centers.

### 2.2 Patient and public involvement statement

No patient involved.

### 2.3 Eligibility criteria

#### 2.3.1 Inclusion criteria

Participants who meet the following criteria will be included: male and female patients aged 18 to 75 years old; fulfill the European League Against Rheumatism (EULAR)/American College of Rheumatology criteria for acute arthritis of gout in 2015; SUA ≥480 μmol/L; or SUA >420 μmol/L, the frequency of acute gout flares in the 12 months before enrollment is ≥2 times/yr, or tophi is present^[10]^; female patients who are underwent sterilization operation or menopause for 2 years.

#### 2.3.2 Exclusion criteria

Potential subjects who meet the inclusion criteria will be excluded if they meet any of the following: patients with acute gout flares in the first 2 weeks of enrollment; colchicine, glucocorticoids, and NSAIDs used within the first 2 weeks of enrollment; allopurinol, probenecid, benzbromarone, and febuxostat tablets or intra-articular injection of glucocorticoid used within the first 2 weeks of enrollment; patients with tophi ulcerated ruptured before enrollment; patients with secondary hyperuricemia caused by kidney disease, hematopathy or some medications, tumor chemoradiotherapy, and organ transplantation; patients with a history of cardiocerebrovascular diseases; patients with a history of peptic ulcer and gastrointestinal bleeding; patients with active stage of liver disease, abnormal liver function or transaminase that is 1.2-fold higher than the upper limit of normality; patients with abnormal renal function, serum creatinine concentration that is 1.2 times higher than the upper limit of normality; patients having malignant tumor or psychosis; patients with allergic or intolerant to febuxostat, colchicine, sinomenine, and any ingredients in the test drug excipients; patients with asthma, urticaria, or other allergies caused by NSAIDs; other (non-gout) chronic arthritis, acute inflammatory arthritis, and autoimmune diseases accompanied by arthritis; those administered with cytotoxic chemotherapeutic drugs.

### 2.4 Who will take informed consent?

The informed written consent from potential trial participants or authorized surrogates will be obtained by a rheumatologist in 7 research centers.

### 2.5 Additional consent provisions for collection and use of participant data and biological specimens

Not applicable.

## 3 Interventions

### 3.1 Explanation for the choice of comparators

A total of 210 patients with gout who meet the study criteria will be randomized (1:1) to the sinomenine group and the colchicine group.

### 3.2 Intervention description

Sinomenine group: sinomenine, 120 mg bid, and colchicine placebo 0.5 mg qd. Colchicine group: sinomenine placebo, 120 mg bid, and colchicine 0.5 mg qd. Both groups will be administered febuxostat (40 mg/d) for ULT. Uniform distribution of medications for gout flares: 120 mg/d of etoricoxib, a one-time distribution for 14 days when the patients are enrolled in the group, and supplements as appropriate in the future; the dosage will be uniformly recorded for the 2 groups. During this trial, all subjects will receive general treatment.

#### 3.2.1 Research procedures

##### 3.2.1.1 Screening

In line with American College of Rheumatology and EULAR, EULAR criteria and the scoring system for acute arthritis of gout in 2015; the eligible patients will be 18 to 75 years old; blood uric acid ≥480 μmol/L; or blood uric acid >420 μmol/L, the frequency of acute gout flares in the 12 months before enrollment of ≥2 times/yr, or with the presence of tophi.

##### 3.2.1.2 Enrollment

Investigators will interview each subject prior to the treatment, and the data will be input into the database along with initial data at the study baseline. The subjects will be divided into test and control groups according to the treatment, and both groups will be treated for 12 weeks. The efficacy and safety of sinomenine in each group before and after treatment during the acute episode of gout lowering uric acid will be observed, respectively.

##### 3.2.1.3 Treatment scheme

Sinomenine group: sinomenine 120 mg bid, colchicine placebo 0.5 mg qd combined with febuxostat 40 mg/d and etoricoxib 120 mg/d for 12 weeks.

Colchicine group: sinomenine placebo 120 mg bid, colchicine 0.5 mg qd combined with febuxostat 40 mg/d and etoricoxib 120 mg/d for 12 weeks.

##### 3.2.1.4 Laboratory indicators

Blood routine, urine routine, liver and renal function, erythrocyte sedimentation rate (ESR), C reactive protein (CRP), uric acid, blood lipids, blood glucose, and 12-lead electrocardiogram.

##### 3.2.1.5 Follow-up

Follow-up will be conducted at baseline and at 2 weeks ± 3 days, 4 weeks ± 3 days, 8 weeks ± 3 days, and 12 weeks ± 3 days.

##### 3.2.1.6 Specimen collection

The following demographic data of the subjects will be collected: age, gender, course of the disease, and history. The subjects will be followed up for 5 times (baseline, 2 weeks, 4 weeks, 8 weeks, and 12 weeks after the treatment) to detect blood routine, urine routine, liver and renal function, ESR, CRP, uric acid, blood lipids, blood glucose, 12-lead electrocardiogram, and record the rate of acute gout flare.

### 3.3 Criteria for discontinuing or modifying allocated interventions

#### 3.3.1 Rejection criteria

(1) The combination of drugs violating the protocol, or patients failing to use drugs in accordance with the provision, which might affect the outcomes.
(2) Incomplete data affecting the efficacy and the judgment of safety.

#### 3.3.2 Shedding criteria

(1) Serious adverse reactions related to the study drug.
(2) Major mistakes that are detected in the clinical research protocol, thus making it difficult to evaluate the efficacy of the drug. Also, a significant deviation observed in the implementation of a well-designed protocol.

#### 3.3.3 Subject termination criteria

(1) Subject terminates spontaneously (e.g., withdrawal of the informed consent);
(2) Subject uses a combination of drugs within a non-prescribed range, which may affect the judgment of efficacy and safety;
(3) Indexes of liver function: transaminase ≥3 times the upper limit of normality for >1 week; indexes of renal function: Cr ≥1.2 times the upper limit of normality for >1 week;
(4) Subject develops adverse events or his condition worsens, leading the investigator to decide that the subject has to quit early;
(5) Other investigators consider that the subject is not suitable to continue and need to quit;
(6) Compliance of the subject to the research protocol is poor, and the quantity and duration of medication do not reach between 80% and 120%.

### 3.4 Strategies to improve adherence to interventions

Subjects will be regularly called back to the hospital for follow-up; they will undergo laboratory tests, and will be asked to return the drug tablet at each follow-up visit.

### 3.5 Relevant concomitant care permitted or prohibited during the trial

This trial will not impose special requirements for care and interventions.

### 3.6 Provisions for post-trial care

When the trial is completed, the subjects will receive standardized treatment based on the guidelines.

### 3.7 Outcomes

#### 3.7.1 Primary outcomes

The rate of acute gout flares in subjects within 12 weeks of the treatment period.

#### 3.7.2 Secondary outcomes

**Figure 1:**
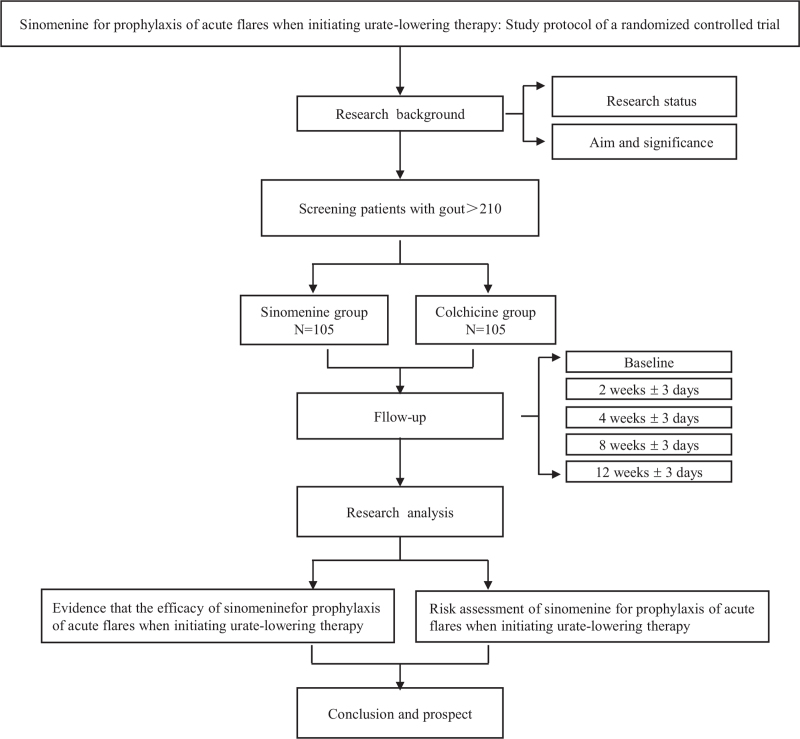
Trial flow and study design.

**Figure 2:**
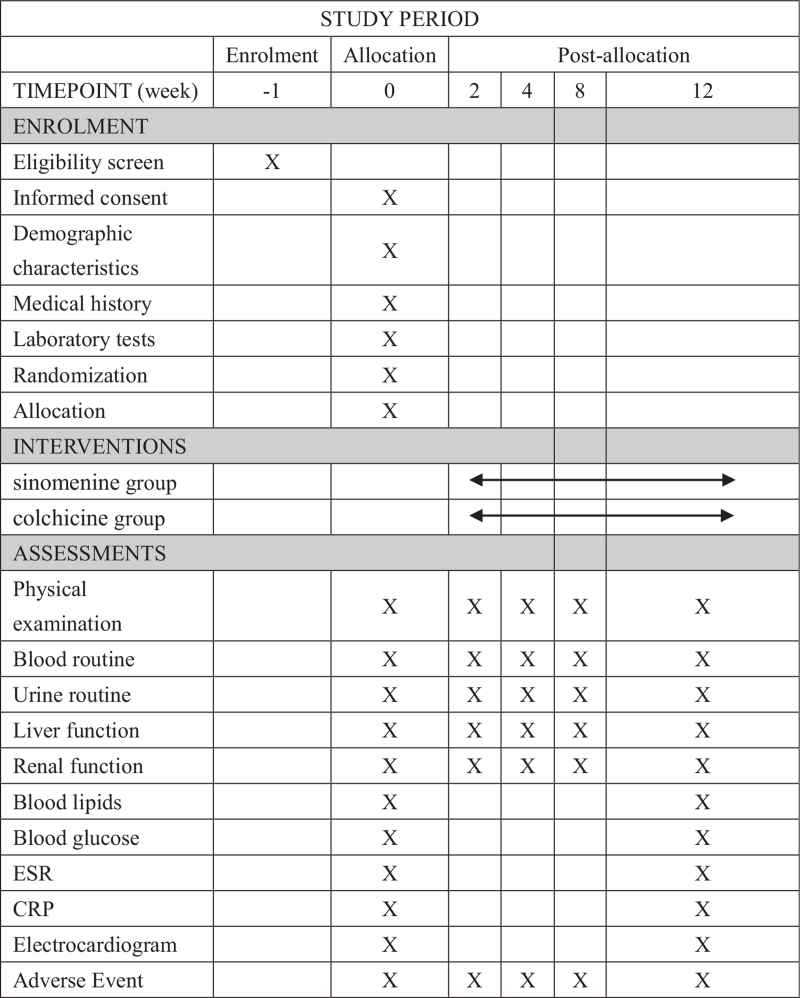
SPIRIT figure of enrollment, interventions, and assessments. Physical examination, blood routine, urine routine, liver and renal function, ESR, CRP, uric acid, blood lipids, blood glucose, 12-lead electrocardiogram. CRP = C reactive protein, ESR = erythrocyte sedimentation rate.

(1) The rate of acute gout flare and the duration of each acute flare of gout within 12 weeks.
(2) The compliance rate of patients whose UA levels are ≤6.0 mg/dL (360 μmol/L) at the weekend of 2nd, 4th, 8th, and 12th week in each group;
(3) The proportion of patients with ≥1 and ≥2 flares of gout within 12 weeks;
(4) Average visual analogue scale/score pain score during gout flares;
(5) The oral dose of etoricoxib will be used to control the onset of acute gout within 12 weeks. Any adverse reactions that occur during the study will be recorded in the “Adverse Reaction Table,” and the patients will be followed up until symptoms disappear or their indicators return to normal. In the event of serious adverse events, necessary measures will be immediately taken to ensure the safety of the subjects. In addition to evaluating the primary and secondary outcomes, safety assessment will be conducted at baseline and 12th week with respect to physical examination, blood routine, urine routine, liver and renal function, ESR, CRP, uric acid, blood lipids, blood glucose, 12-lead electrocardiogram (Figs. 1 and 2).

### 3.8 Participant timeline

Figure 2.

### 3.9 Sample size

This is a non-inferiority test. The primary outcome will be the rate of acute gout flares in subjects within 12 weeks of the treatment period. According to the research hypothesis, the test level is a 2-sided *P*-value of .05 and power of 80%, while the ratio of the test group to the control group is 1:1. The parameters of the preliminary study indicated that the rate of gout flares in the control group was 19% within 12 weeks, so it was estimated that the test group was 15%. The estimated sample size for each group was 89, with a total of 178 participants for the 2 groups. In this clinical trial, the shedding rate did not exceed 15%, and the sample size for each group was determined to be 105 per group, totaling 210.

### 3.10 Recruitment

The randomized controlled trial (RCT) that is blinded to assessors and patients will be conducted at 7 research centers. The subjects in this study will be recruited from outpatient and inpatient wards by placing advertisements on social media platforms of the hospital departments and distributing posters in public areas of the hospital with details of the study and contact information.

Patients who meet the criteria will be invited to participate in the study, and they will be provided with the details of the RCT protocol. All subjects will be required to sign an informed consent and provide complete information. The subjects will be randomized (1:1) to the test group and the control group. None of the patients will know about their assigned group in order to keep the trial blind. Then, the baseline characteristics will be assessed and a database record will be obtained by the same physician. All subjects will be treated for 12 weeks and followed up on 2nd, 4th, 8th, and 12th weeks, after which the data will be evaluated.

In addition, all the subjects will be given a schedule of intervention dates and follow-up appointments. These evaluations will be performed by an assistant who will be involved neither in the randomization nor in the treatment.

## 4 Assignment of interventions: allocation

### 4.1 Sequence generation

The statistician will use the PROC PLAN process of SAS 9.4 statistical software to generate random numbers with block randomization of variable block length.

### 4.2 Concealment mechanism

The central randomization was used. The random number and the drug number are unified.

### 4.3 Implementation

We implement random masking through double-blind and double-simulation design of drugs. When the subjects are allocated, the random numbers are obtained through the central random system. Researchers and subjects only know the drug number, so as to avoid the destruction of random grouping.

## 5 Assignment of interventions: blinding

### 5.1 Who will be blinded

A total of 210 eligible subjects will be randomly assigned to 2 parallel groups. Statisticians will adopt the PROC PLAN process of SAS 9.4 statistical software to generate a random list by randomization of block size of 4. Then, the clinical trial manager will divide them into 2 groups according to the random list. All people involved including the investigators will be blinded to the assignment of the subjects. The placebo will be similar to sinomenine in size, weight, shape, and color. All study site personnel, the subject, the sponsor, and the Contract Research Organization (CRO) will remain blinded to the study.

### 5.2 Procedure for unblinding if needed

First, unblinding meetings will be held. Unblinding in emergency situations is also implemented through the central random system.

## 6 Data collection and management

### 6.1 Plans for assessment and collection of outcomes

The Case Report Form will be used to assess and collect outcomes and baseline. In addition, the clinical trial database will be constructed by a designated data manager responsible for regular database management and maintenance. All data will be imported into the clinical trial database by 2 research assistants. The investigator will be responsible for maintaining accurate, complete, and up-to-date records for each subject. The investigator will also be responsible for maintaining any source documentation related to the study, including any films, tracings, computer discs, or tapes.

The anonymity of participating subjects will be maintained. For data collection and management purposes, subjects will only be identified by a subject number. Documents that identify the subject beyond subject number will not be submitted to the sponsor (e.g., the signed informed consent document; subject initials) and will be maintained in strict confidence by the investigator, except to the extent necessary to allow auditing by the regulatory authorities, study monitor, or sponsor representatives.

Site personnel will record all data for each study subject through electronic case report forms (eCRFs) using an Electronic Data Capture (EDC) system provided and approved by the sponsor. Study Procedures Manual for additional information regarding CRFs will be used as source documentation. Sites will complete the eCRFs in a timely manner, and the investigator will promptly review the completed eCRFs after every visit for each subject. As the person ultimately responsible for the accuracy of all eCRF data, the investigator will sign the Investigator's Statement in each subject's eCRF.

### 6.2 Plans to promote participant retention and complete follow-up

Follow-up will be conducted at baseline, at 2 weeks ± 3 days, 4 weeks ± 3 days, 8 weeks ± 3 days, and 12 weeks ± 3 days. Any missing or incorrect data will be detected by the software system. In such case, the original CRFS will be checked to correct or complete every piece of data.

### 6.3 Data management

The clinical trial database is constructed by a designated data manager who will be responsible for the regular database management and maintenance. All data will be imported into the clinical trial database by 2 research assistants. The EDC system automatically generates queries resulting from the computer checks embedded into the system, so as to ensure accuracy, quality, consistency, and completeness of the database. Manual queries resulting from review by monitors, medical coders, and other Data Management staff will also be generated from within the EDC system, where they are tracked. Sites can resolve the queries and correct the entered data when necessary. Every change to data will be captured in the EDC system audit trail. Upon completion of the study, or after reaching a pre-specified point in the study, Data Management will lock the database and generate the SAS datasets necessary for data analysis and reporting.

### 6.4 Confidentiality

The confidentiality measures are as follows. The results of this research project may be published in medical journals. The subject's information will be represented by a unique number, and the coded information will be stored in the school of Public Health, Fujian Medical University. The information of subjects will be maintained confidential as required by law. However, records of subjects may be reviewed to ensure that the study complies with applicable laws and regulations.

### 6.5 Plans for collection, laboratory evaluation, and storage of biological specimens for genetic or molecular analysis in this trial/future use

Not applicable.

## 7 Statistical methods

### 7.1 Statistical methods for primary and secondary outcomes

The primary outcomes will be compared between the groups using Pearson chi-square test. t-Test, corrected t-test (equal variance not assumed), and analysis of variance for repeated measurements will be used for data analysis, and the grade data will be assessed by Wilcoxon 2-sample test.

### 7.2 Interim analyses

Doctors will have access to these interim results and make the final decision to terminate the trial.

### 7.3 Methods for additional analyses

The hybrid control will use multivariate logistic regression, estimating the odds ratio and 95% confidence interval. Clinically significant variables from the univariate analysis will be included in the multivariate model. The goodness of fit will be evaluated by Hosmer–Lemeshow test. Statistical analysis will be carried out using SPSS (version 26; SPSS Inc., Chicago, IL, USA). A 2-tailed significance level of 0.05 will be used for all tests. *P* < .05 will indicate statistical significance.

### 7.4 Methods in analysis to handle protocol non-adherence and any statistical methods to handle missing data

Study populations include the intent-to-treat analysis set defined as all randomized patients and the per-protocol analysis set defined as all patients in the intent-to-treat population without any major protocol deviations. Multiple imputations will be used to manage missing values.

### 7.5 Plans to give access to the full protocol, participant level-data, and statistical code

Data are available upon reasonable request. All inquiries about data sharing will be available at: sailing1980@126.com.

## 8 Oversight and monitoring

### 8.1 Composition of the coordinating center and trial steering committee

The Steering Committee consists of 3 members with 2 senior rheumatologists and a statistician who will supervise the trial and ensure the safety and quality of data. The committee is independent of the research team and has no conflict of interest with the investigators. The committee will provide regular supervision, hold monthly meetings, and organize a field trip at least once to ensure the trial is carried out smoothly and ethically. Also, a supervisor will ensure the authenticity and integrity of the data. During the visit, they will interview the investigators, check the original research documents and the registration of subjects, and confirm whether the clinical center complies with the research protocol. Any non-compliance with the agreement will be fully recorded using a violation report form. Furthermore, they will also identify the problems in the trial and put forward suggestions on the modification of the protocol. If any decision to amend the protocol has to be made, the approval will be sent to the Institutional Medical Ethics Committee in writing, and the investigator will be notified in writing after approval. The protocol will be immediately updated in the system.

### 8.2 Composition of the data monitoring committee, its role, and reporting structure

The data monitoring committee will include 3 members with EDC system administrator, data administrator, and statistician. The committee is independent of the research team and has no conflict of interest with the investigators. They will carry out data management design, processing and regulation, quality control, security and confidentiality measures, and EDC system management.

### 8.3 Adverse event reporting and harms

#### 8.3.1 Observation and recording

Any adverse reaction during the study period will be filled in the “adverse event form,” and follow-up investigation will include detailed records of the treatment process and results, until the laboratory examination returns to normal, the symptoms and signs disappear.

#### 8.3.2 Medical treatment

When an adverse reaction is discovered, the investigator will decide on the diagnosis and treatment measures based on the condition, and will also decide whether to terminate the observation. In the event of a serious adverse event, the unit undertaking the clinical research will immediately take necessary measures to protect the safety of the subjects.

#### 8.3.3 Report of adverse events

The investigator will evaluate each adverse event, and if a serious adverse event occurs, it will be reported in accordance with the serious adverse event reporting requirements.

All adverse events will be recorded in the adverse event page of the medical record report form. For each adverse event, the duration, severity, and test drug will be described, including the relevance, and necessity to take measures as well as the taken treatments.

#### 8.3.4 Requirements for reporting serious adverse events

If a serious adverse event occurs, the investigator will take appropriate treatment/rescue measures for the subject according to his own clinical judgment to protect the safety of the subject.

The investigator should notify the main investigator and the ethics committee of the clinical research institution by the fastest means of communication within 24 hours after learning of the serious adverse event. The investigator will submit the completed serious adverse event report form to the lead unit and the main investigator, who will file it to the team leader's ethics committee within 3 days after being informed.

### 8.4 Frequency and plans for auditing trial conduct

The trial conduct will be audited every 2 months.

### 8.5 Plans for communicating important protocol amendments to relevant parties (e.g., trial participants, ethical committees)

Committees will identify the problems in the trial and put forward suggestions on the modification of the protocol. If any decision to amend the protocol is to be made, a written application is to be submitted to the Institutional Medical Ethics Committee, and the investigator will be notified in writing after approval. The protocol will be immediately updated in the system.

### 8.6 Dissemination plans

All presentations will protect the integrity of the primary research objectives. None of the data that compromise the blinding will be released before the results are available. The Steering Committee will discuss the recommendations on the timing of these final data that may be presented at the meetings. The primary outcomes will be published in abstract books and articles.

## 9 Discussion

Besides long-term ULT that can achieve the target serum urate level for gout, there are also anti-inflammatory therapies for acute flares and adequate anti-inflammatory prophylaxis.^[11]^ Sinomenine is an alkaloid derived from the Chinese herb sinomenium acutum, whose anti-inflammation effects have been widely evaluated and confirmed. Sinomenine has been used in a series of proprietary Chinese medicines for treating rheumatoid arthritis and other autoimmune diseases due to its anti-inflammatory and immunosuppressive properties.^[12]^ Herein, we have designed a RCT protocol to compare the clinical efficacy of sinomenine and colchicine for prophylaxis of acute flares when initiating urate-lowering therapy. In addition to laboratory tests, we mainly plan to observe the frequency of gout flares and the visual analogue scale/score pain score so as to evaluate the efficacy of sinomenine for prophylaxis of acute flares when initiating urate-lowering therapy. When gout patients also suffer from kidney disease, cardiovascular disease, diabetes, and other diseases, the curative effect of sinomenine may provide a new solution by overcoming the restricted use of colchicine, non-steroidal anti-inflammatory drugs, or glucocorticoids to prevent gout flares.

The present study has some limitations. First, the universality of the sample population and the research centers involved in the trial is limited. Although the multicenter selection of this experiment can increase the number of participants, the situation in each center is complicated and long-distance communication is difficult, which may affect the progress of the group. Secondly, the difficulties in the study include the subjects’ timely visits, poor compliance, and loss of follow-up caused by taking drugs in violation of the protocol, and the accuracy of the number of pain records during the subject's research. It should be noted that there are some reports on the possible side effects of sinomenine. If the participant experiences serious adverse reactions such as allergies, the investigator will immediately take treatment measures.

## 10 Trial status

The revised version V20210830 of this protocol on August 30, 2021, has been approved by the Institutional Medical Ethics Committee of Shenzhen Traditional Chinese Medicine Hospital, Southern Hospital of Southern Medical University, Guangdong Provincial Hospital of Traditional Chinese Medicine, First Affiliated Hospital of Tianjin University of Traditional Chinese Medicine, People's Hospital of Longhua District Shenzhen, Second Affiliated Hospital of Guizhou University of Traditional Chinese Medicine, and Henan Provincial Hospital of Traditional Chinese Medicine. The recruitment began on September 1, 2021, and the study will be completed on March 30, 2023.

## Acknowledgments

The authors would like to thank Dr Zhiying Zhan for the help in the sample size estimation and statistical analysis methods.

## Author contributions

Ertao Jia and Jianyong Zhang designed the randomized placebo-controlled trial. Ertao Jia, Shasha Hu, and Hongling Geng drafted the manuscript. Haiqiong Zhu, Jingjing Xie, Yuya Xiao, Yubao Jiang, and Min Xiao conducted the research. Ertao Jia was responsible for the statistical analyses. All authors participated in the manuscript revision.

**Investigation:** Jingjing Xie.

**Project administration:** Ertao Jia.

**Resources:** Min Xiao, Yubao Jiang, Yuya Xiao.

**Supervision:** Jianyong Zhang.

**Writing – original draft:** Ertao Jia, Shasha Hu, Hongling Geng, Haiqiong Zhu.
